# β-lapachone-mediated WST1 Reduction as Indicator for the Cytosolic Redox Metabolism of Cultured Primary Astrocytes

**DOI:** 10.1007/s11064-023-03878-z

**Published:** 2023-02-22

**Authors:** Patrick Watermann, Ralf Dringen

**Affiliations:** 1 Centre for Biomolecular Interactions Bremen, Faculty 2 (Biology/Chemistry), University of Bremen, P.O. Box 330440, 28334 Bremen, Germany; 2 Centre for Environmental Research and Sustainable Technologies, University of Bremen, Bremen, Germany

**Keywords:** Astrocytes, Glucose, Metabolism, NADPH, NQO1, Pentose-phosphate Pathway

## Abstract

Electron cycler-mediated extracellular reduction of the water-soluble tetrazolium salt 1 (WST1) is frequently used as tool for the determination of cell viability. We have adapted this method to monitor by determining the extracellular WST1 formazan accumulation the cellular redox metabolism of cultured primary astrocytes via the NAD(P)H-dependent reduction of the electron cycler β-lapachone by cytosolic NAD(P)H:quinone oxidoreductase 1 (NQO1). Cultured astrocytes that had been exposed to β-lapachone in concentrations of up to 3 µM remained viable and showed an almost linear extracellular accumulation of WST1 formazan for the first 60 min, while higher concentrations of β-lapachone caused oxidative stress and impaired cell metabolism. β-lapachone-mediated WST1 reduction was inhibited by the NQO1 inhibitors ES936 and dicoumarol in a concentration-dependent manner, with half-maximal inhibition observed at inhibitor concentrations of about 0.3 µM. β-lapachone-mediated WST1 reduction depended strongly on glucose availability, while mitochondrial substrates such as lactate, pyruvate or ketone bodies allowed only residual β-lapachone-mediated WST1 reduction. Accordingly, the mitochondrial respiratory chain inhibitors antimycin A and rotenone hardly affected astrocytic WST1 reduction. Both NADH and NADPH are known to supply electrons for reactions catalysed by cytosolic NQO1. Around 60% of the glucose-dependent β-lapachone-mediated WST1 reduction was prevented by the presence of the glucose-6-phosphate dehydrogenase inhibitor G6PDi-1, while the glyceraldehyde-3-phosphate dehydrogenase inhibitor iodoacetate had only little inhibitory potential. These data suggest that pentose phosphate pathway-generated NADPH, and not glycolysis-derived NADH, is the preferred electron source for cytosolic NQO1-catalysed reductions in cultured astrocytes.

## Introduction

The quinone β-lapachone has various promising pharmacological activities which makes it interesting as potential drug in cancer therapy and for anti-bacterial and anti-parasite treatments [1–3]. In cells, β-lapachone is reduced by NAD(P)H: quinone acceptor oxidoreductase 1 (NQO1, EC 1.6.99.2) by a two-electron reduction process to its hydroquinone β-lapachol [4]. However, the generated hydroquinone is labile and autoxidises quickly in two distinct one-electron steps, thereby generating superoxide which can subsequently damage tumour cells that are known to express high activities of NQO1 [5]. However, as also brain astrocytes contain substantial activity of NQO1 [6] such normal body cells have to be considered as potential unwanted target of a β-lapachone treatment [7].

In brain, astrocytes have a strategically important location between capillaries and other parenchymal cells, thereby regulating the exchange of substances between blood and brain [8]. In addition, astrocytes have a plethora of important functions in brain metabolism, in the defence against oxidative stress and toxins, in the homeostasis of the extracellular environment and they contribute also to the development and maintenance of cognitive functions [9–13].

So far only a few studies report consequences of an exposure of astrocytes to β-lapachone. Low micromolar concentrations of β-lapachone for extended incubation periods have been reported to improve the antioxidative potential of cultured astrocytes [14]. In addition, submicromolar concentrations of β-lapachone have been shown to activate glutamate dehydrogenase and to counteract iodoacetate induced toxicity in cultured astrocytes [15]. In contrast, in concentrations above 10 µM β-lapachone causes acute ROS formation, glutathione disulfide (GSSG) accumulation, metabolic impairment and toxicity in cultured astrocytes [7]. All these adverse effects of a treatment with high micromolar concentrations of β-lapachone are abolished in the presence of the NQO1 inhibitor dicoumarol, demonstrating that the reduction of β-lapachone by NQO1 is exclusively responsible to initiate the adverse consequences of an acute β-lapachone treatment [7].

NQO1 is a cytosolic oxidoreductase that can accept electrons from both NADH and NADPH as electron source [6, 16]. The cellular potential for NQO1-mediated intracellular reduction can be monitored by quantifying the extracellular reduction of the water-soluble tetrazolium salt (WST1) to its formazan, if the NQO1 is reducing as substrate a redox cycler that transports electrons from the intracellular compartment to the extracellular space [17]. This allows the investigation of intracellular metabolic pathways that can provide NAD(P)H for the NQO1 reduction by monitoring extracellular WST1 reduction. Menadione has been used as redox cycler for such studies [6, 17], but also β-lapachone has the potential to mediate as redox cycler NQO1-dependent extracellular WST1 formazan generation [7]. However, both redox cyclers have been reported in higher concentrations to cause oxidative stress in astrocytes [7, 18, 19].

β-lapachone in a concentration of 20 µM has recently also been reported to act in cultured astrocytes as electron cycler that transfers in its reduced β-lapachol form electrons derived from intracellular NQO1-dependent reduction through the membrane to allow extracellular WST1 reduction [7]. However, such high concentrations of β-lapachone induce oxidative stress [7] which has been demonstrated to affect metabolic pathways such as glycolysis and pentose-phosphate pathway which contribute to the regeneration of NADH and NADPH [7, 19–21]. Therefore, we have investigated in the current study whether also low micromolar concentrations of β-lapachone could be suitable to act as electron cycler to monitor cellular NAD(P)H regeneration without causing intracellular oxidative stress, alterations in metabolism and cell damage. Here we show that low concentrations of up to 3 µM β-lapachone allow to monitor intracellular NAD(P)H generating metabolic processes via extracellular WST1 reduction without causing any obvious adverse effects. Data obtained by the optimised test system revealed that mitochondrial metabolic pathways are hardly involved in providing electrons for NQO1-mediated β-lapachone reduction and that pentose-phosphate pathway (PPP)-derived NADPH serves as prominent electron source for NQO1-dependent reduction processes in cultured astrocytes.

## Materials and Methods

### Materials

Dulbecco’s modified Eagle’s medium (DMEM with 25 mM glucose) powder and penicillin/streptomycin solution was obtained from Gibco/Invitrogen (Darmstadt, Germany) and fetal calf serum (FCS) from Sigma-Aldrich (Steinheim, Germany). β-lapachone (ab141097) was purchased from Abcam (Berlin, Germany), dicoumarol (M1390) and iodoacetate (I6375) were from Sigma-Aldrich (Steinheim, Germany). ES936 (sc-362737) was obtained from Santa Cruz Biotechnology (Heidelberg, Germany), WST1 (W201) from Dojindo (Munich, Germany) and G6PDi-1 (31484) from Cayman Chemical (Tallinn, Estonia). The enzymes for the metabolite assays were purchased from Roche Diagnostics (Mannheim, Germany). All other chemicals of the highest purity available were purchased from Merck (Darmstadt, Germany), Sigma-Aldrich (Steinheim, Germany) or AppliChem (Darmstadt, Germany). Sterile cell culture material and non-sterile microtiter plates were from Sarstedt (Nümbrecht, Germany).

### Astrocyte-rich Cultures

Wistar rats were obtained from Charles River Laboratories (Sulzfeld, Germany). Animals were treated in accordance with the animal regulations of the University of Bremen and of the State of Bremen, Germany. Primary astrocyte-rich cultures were prepared from whole brains of new-born Wistar rats following the protocol previously described [22]. The brains were mechanically dissociates as described and 300,000 harvested cells were seeded in 1 mL of culture medium (90% DMEM, 10% FCS, 1 mM pyruvate, 18 U/mL penicillin G and 18 µg/mL streptomycin sulfate) in wells of a 24well plate. The cultures were incubated for at least two weeks at 37 °C with 10% CO_2_ in the humidified atmosphere of a cell incubator (Sanyo, Japan). The medium was renewed every 7 days and one day before an experiment. Experiments were performed on confluent astrocyte cultures of an age between 14 and 36 days.

### Experimental Incubations

**Fig. 1:**
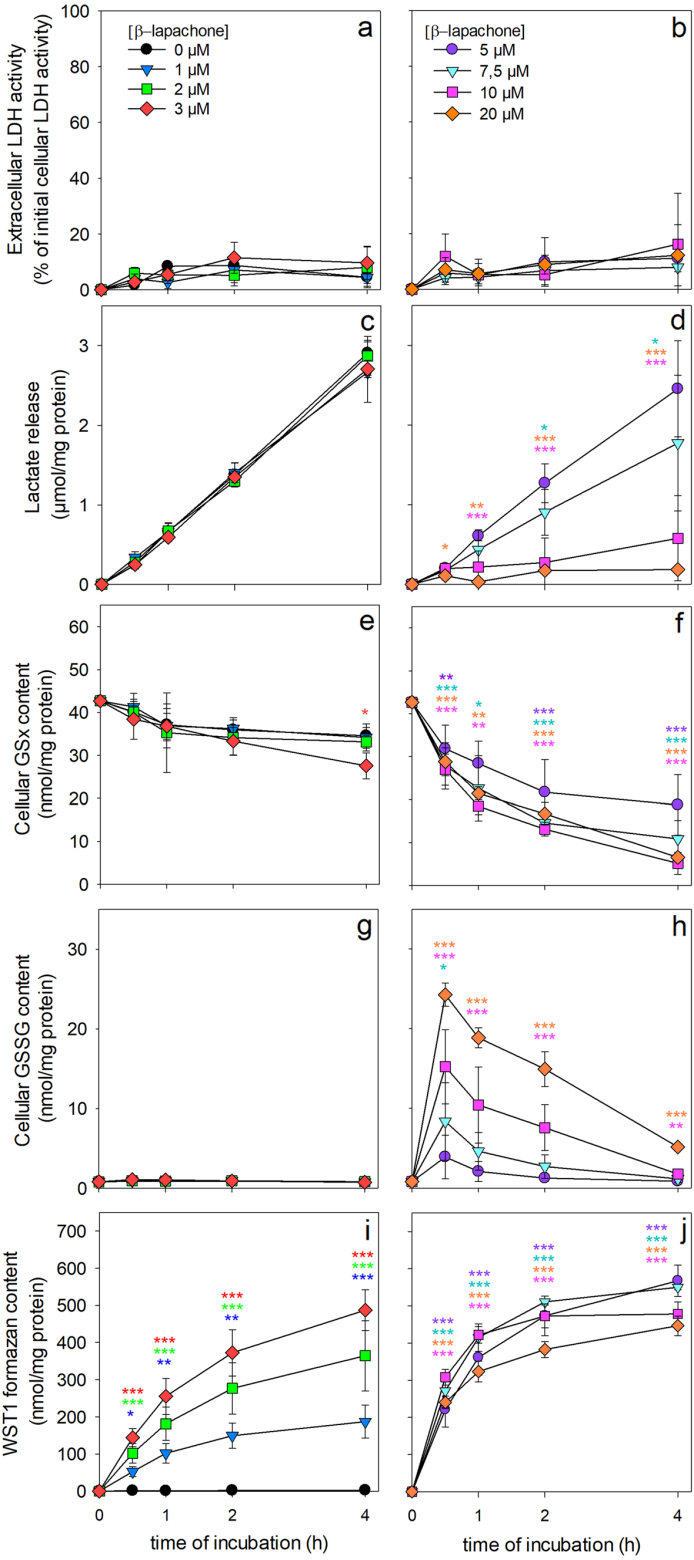
Time- and concentration-dependent effects of βlapachone on the viability, the lactate release, the cellular GSx and GSSG contents and the WST1 reduction in cultured primary astrocytes. The cells were incubated without (0 µM) or with the indicated concentrations of β-lapachone in the presence of 400 µM WST1 in 200 µL glucose-containing incubation buffer (5 mM) for up to 4 h. Extracellular LDH activity (**a**, **b**), lactate release (**c**, **d**), cellular GSx (**e**, **f**) and GSSG contents (**g**, **h**) as well as the extracellular WST1 formazan content (**i**, **j**) were determined for the indicated time points. The initial specific GSx content of the cells was 43.0 ± 0.6 nmol/mg protein and the specific initial GSSG content was 1.0 ± 0.1 nmol GSx/mg protein. The initial protein content was 108 ± 2 µg per well. The data presented are means ± SD of values from experiments that had been performed on three independently prepared astrocyte cultures. The significance of differences (as calculated by ANOVA) compared with data for incubations without β-lapachone (0 µM) is indicated by *p < 0.05, **p < 0.01, or ***p < 0.001 in the colours of the symbols indicating the β-lapachone concentrations applied

If not stated otherwise, confluent astrocyte cultures were washed twice with 1 mL pre-warmed (37 °C) incubation buffer (IB; 20 mM HEPES, 145 mM NaCl, 5.4 mM KCl, 1.8 mM CaCl_2_, 1 mM MgCl_2_, 0.8 mM Na_2_HPO_4_, pH 7.4) and incubated at 37 °C with 400 µL IB that contained 400 µM WST1 and 3 µM β-lapachone and had been supplemented with glucose and/or the NQO1 inhibitors ES936 or dicoumarol in concentrations of up to 3 µM as indicated in the figure legends in the humidified atmosphere (without CO_2_) of a cell incubator (Sanyo, Japan). For some experiments (indicated in the legends of the figures), the cells were washed and pre-incubated in 400 µL glucose-free IB for 30 min to deprive them of glycolysis substrates and metabolic intermediates before the main incubation in 400 µL buffer was started. Extracellular substrates for astrocytic metabolism were applied for the main incubation in concentrations of 5 mM, while metabolic inhibitors were applied, if indicated, in final concentrations of 10 µM (antimycin A, BAM-15, G6PDi-1 and rotenone) or 0.3 mM (iodoacetate). To obtain the data shown in Figs. 1 and 5, the cells were washed twice with 1 mL pre-warmed (37 °C) IB and incubated for the time periods given with 200 µL IB that contained 400 µM WST1, 5 mM glucose and the concentrations of substances indicated in the figure legends. After the given incubation periods, media samples were harvested for determination of extracellular contents of WST1 formazan and lactate as well as the activity of extracellular lactate dehydrogenase (LDH). The cells were washed with 1 mL ice-cold (4 °C) phosphate-buffered saline (PBS; 10 mM potassium phosphate buffer, pH 7.4, containing 150 mM NaCl) and lysed for analysis of the contents of total glutathione (GSx) and glutathione disulfide (GSSG), the content of cellular protein and the initial LDH activity.

**Fig. 2:**
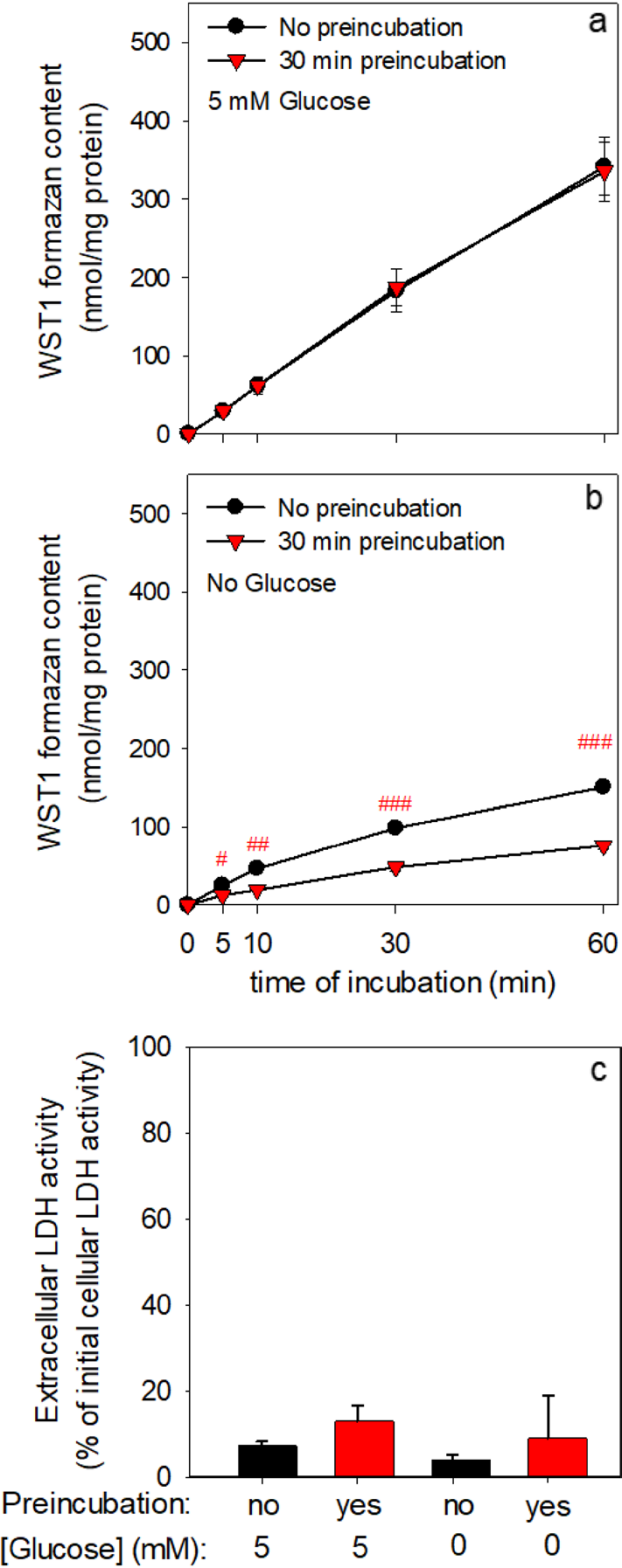
Test for glucose-independent β-lapachone-mediated WST1 reduction by cultured astrocytes. Cells were either pre-incubated for 30 min without glucose or not preincubated before the incubation was started in 400 μL IB containing 400 μM WST1 plus 3 μM β-lapachone in the presence (**a**) or in the absence (**b**) of 5 mM glucose. For the indicated time points (**a**, **b**) or for the 60 min incubation (**c**) the extracellular content of WST1 formazan (**a**, **b**) and the extracellular LDH activity (**c**) were determined. The initial protein content of the astrocyte cultures was 119 μg ± 20 μg per well. The data represent mean values ± SD of data from experiments on three independently prepared astrocyte cultures. The significance of differences (as calculated by paired t-test) is indicated by ^#^p < 0.05, ^##^p < 0.01, or ^###^p < 0.001.

### Determination of Extracellular Contents of WST1 Formazan

The water-soluble tetrazolium salt 1 (WST1) is a membrane impermeable substance that can be reduced in cell cultures by membrane permeable electron cyclers such as β-lapachone to form the water-soluble WST1 formazan [7, 17]. The extracellular content of WST1 formazan in the harvested media samples was determined by photometric determination as previously described [17]. Depending on the content of WST1 formazan, media samples of 25 µL or 50 µL were diluted with water to a total volume of 200 µL in wells of a microtiter plate and the absorbance of the WST1 formazan generated was measured at 450 nm in a microtiter plate reader (Multiscan Sky, Thermo Fisher, Darmstadt, Germany). The concentration of WST1 formazan in the incubation media was calculated from the absorbance by using the extinction coefficient of 35.2 mM^−1 ^× cm^−1^ [17]. During the incubations in 400 µL buffer for determination of the extracellular WST1 generation 50 µL of media were taken out at the indicated time points. The total amounts of WST1 formazan generated were corrected for the amounts present in the samples that had been taken out to determine WST1 formazan amounts generated at earlier time points.

### Test for Cell Viability and Determination of Protein Content

The viability of astrocyte cultures during or after a given treatment was determined by testing for metabolic lactate generation and for a potential release of the cytosolic enzyme LDH. Quantification of extracellular lactate content and of extracellular LDH activity was done by photometric microtiter plate assays as described previously in detail [22]. For incubations in 400 µL IB all values were corrected for the amounts present in the samples that had been taken out to obtain data for earlier time points. The initial protein content of the astrocyte cultures used for the experiments was determined according to the Lowry method [23] using bovine serum albumin as standard protein.

### Quantification of Glutathione and Glutathione Disulfide

The contents of cellular and extracellular total glutathione (GSx = amount of glutathione (GSH) plus twice the amount of GSSG) and GSSG were quantified by a colorimetric enzymatic cycling assay as previously described in detail [22] which represent a modification of the method originally described by Tietze [24].

### Data and Statistical Analysis

The data presented are means ± standard deviations (SD) of values that are derived from experiments performed on at least three independently prepared astrocyte cultures. The significance of differences between two data sets were analysed with a t-test and three or more data sets were analysed by ANOVA followed by a Bonferroni *posthoc* test. The level of significance is indicated by hashes or asterisks (^#^or *p < 0.05, ^##^or **p < 0.01, ^###^or ***p < 0.001). p-values above 0.05 were considered as not significant.

## Results

### Test for Potential Adverse Consequences of an Exposure of Cultured Astrocytes to Low Micromolar Concentrations of β-lapachone

To test for potential adverse consequences of low micromolar concentrations of β-lapachone in the presence of WST1, cultured astrocytes were exposed to β-lapachone in concentrations of up to 20 µM for up to 4 h and the cell viability (Fig. 1a, b), lactate release (Fig. 1c, d), cellular GSx (Fig. 1e, f) and GSSG (Fig. 1g, h) contents and WST1 reduction (Fig. 1i, j) were determined. Incubation of astrocytes with up to 20 µM β-lapachone for 4 h did not cause any increase in the extracellular LDH activity (Fig. 1a, b). In contrast, concentration-dependent modifications of the other parameters investigated were found. β-lapachone in concentrations of up to 3 µM did within 4 h of incubation not affect extracellular lactate accumulation (Fig. 1c) and at best slightly the cellular GSx content (Fig. 1e) nor caused this concentration any detectable cellular GSSG accumulation (Fig. 1g). In contrast, concentrations of β-lapachone above 3 µM lowered lactate production (Fig. 1d), caused a significant loss in cellular GSx content (Fig. 1f) and caused rapid accumulation of GSSG in the cells (Fig. 1h). In the presence of β-lapachone a rapid increase in the extracellular content of WST1 formazan was observed that depended strongly on the concentration of β-lapachone applied to the cells and slowed down after the initial 60 min of incubation. The WST1 reduction in the presence of up to 3 µM β-lapachone was almost proportional to the applied concentration of β-lapachone (Fig. 1i), while higher β-lapachone concentrations caused a very rapid increase in extracellular WST1 formazan content but did not lead to higher total WST1 formazan levels (Fig. 1j) compared to the incubation with 3 µM β-lapachone (Fig. 1i).

These results revealed that β-lapachone in concentrations of up to 3 µM does not compromise cell viability and glycolytic lactate formation in cultured astrocytes nor causes substantial oxidative stress that would be detectable by an increase in cellular GSSG. As in the presence of 3 µM β-lapachone a strong extracellular WST1 formazan accumulation was observed, the concentration of 3 µM β-lapachone was chosen as redox cycler for further experiments to monitor cellular metabolism by β-lapachone-dependent WST1 reduction in viable astrocytes. Control experiments that tested for a potential astrocytic WST1 reduction in the absence of β-lapachone under the conditions used clearly demonstrated that extracellular formation of WST1 formazan was not detectable in the absence of β-lapachone (Fig. 1i), confirming literature data reporting that an electron cycler is required to mediate the transfer of electrons from intracellular NAD(P)H for extracellular WST1 reduction [17].

### WST1 Reduction by Astrocytes in the Absence or the Presence of Glucose

To test for the need of glucose as metabolic substrate for metabolic processes that provide the NAD(P)H required for NQO1-dependent β-lapachone reduction, astrocytes were incubated without or with 5 mM glucose in the presence of β-lapachone and WST1. For cells exposed to β-lapachone and WST1 in the presence of glucose, an almost linear extracellular accumulation of WST1 formazan was observed that accounted to around 340 nmol/mg after 60 min of incubation (Fig. 2a). A slower increase in extracellular WST1 accumulation was also observed for cells that had been incubated without glucose. This condition yielded an extracellular WST1 formazan content of around 150 nmol/mg within 60 min of incubation (Fig. 2b). To deplete astrocytes of intracellular glycogen, free glucose and glycolytic intermediates before the main incubation was started, the cultures were preincubated for 30 min in glucose-free IB. Such a preincubation did not affect the cell viability (Fig. 2c) nor the WST1 reduction in glucose-exposed cultures (Fig. 2a), but lowered significantly the rate of WST1 formazan accumulation during incubation in the absence of glucose (Fig. 2b).

**Fig. 3:**
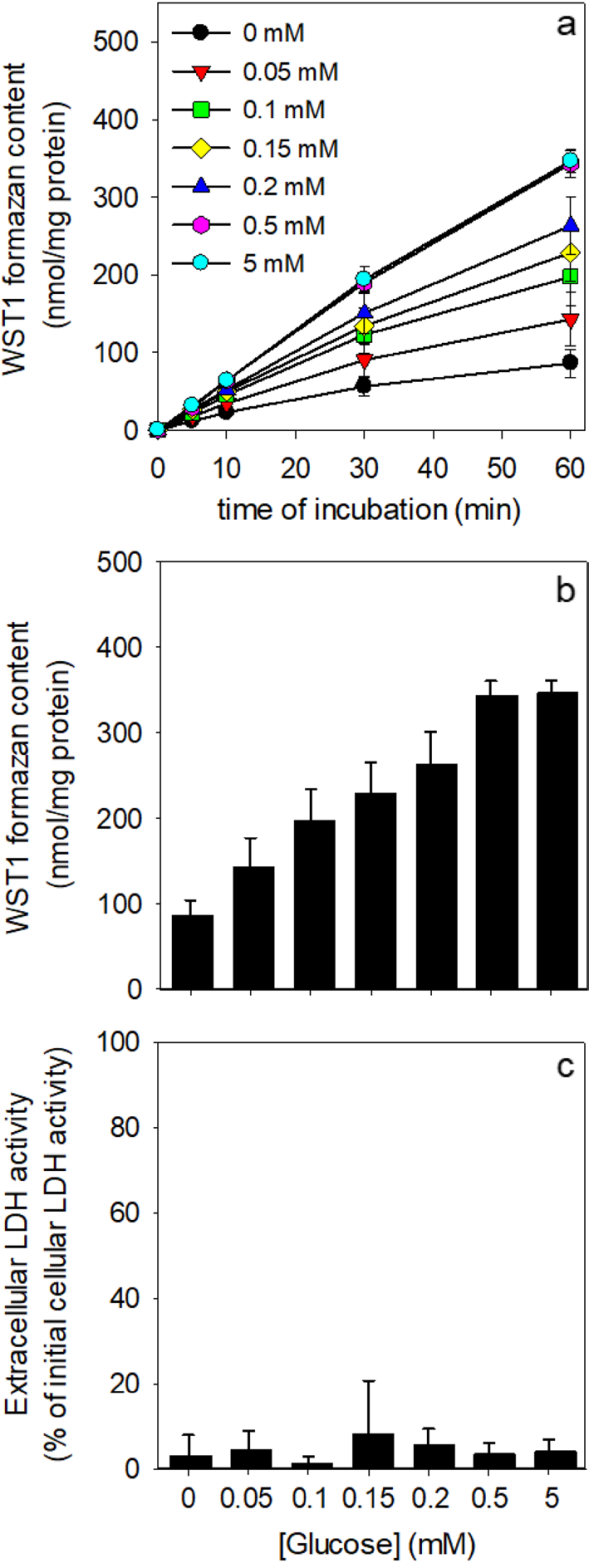
Glucose dependency of the β-lapachone-mediated WST1 reduction by cultured astrocytes. The cells were pre-incubated for 30 min in glucose-free IB and subsequently incubated in 400 µL IB without (0 µM) or with the indicated concentrations of glucose in the presence of 400 µM WST1 and 3 µM β-lapachone. Extracellular WST1 formazan content (**a**, **b**) and extracellular LDH activity (**c**) were determined for the indicated time points **a** or after the 60 min incubation **b**, **c**. The initial protein content was 133 ± 4 µg per well. The data shown are means ± SD of results obtained in experiments on three independently prepared astrocyte cultures

The low rate of glucose-independent WST1 reduction after preincubation in glucose-free buffer (Figs. 2b and 3a) was strongly increased by application of glucose in a concentration-dependent manner (Fig. 3a, b). Half-maximal extracellular accumulation of WST1 formazan within 60 min of incubation was found for an initial glucose concentration of around 0.1 mM (Fig. 3b). Neither the preincubations nor the main incubations with or without glucose caused any significant loss in cell viability (Figs. 2c and 3c).

**Fig. 4:**
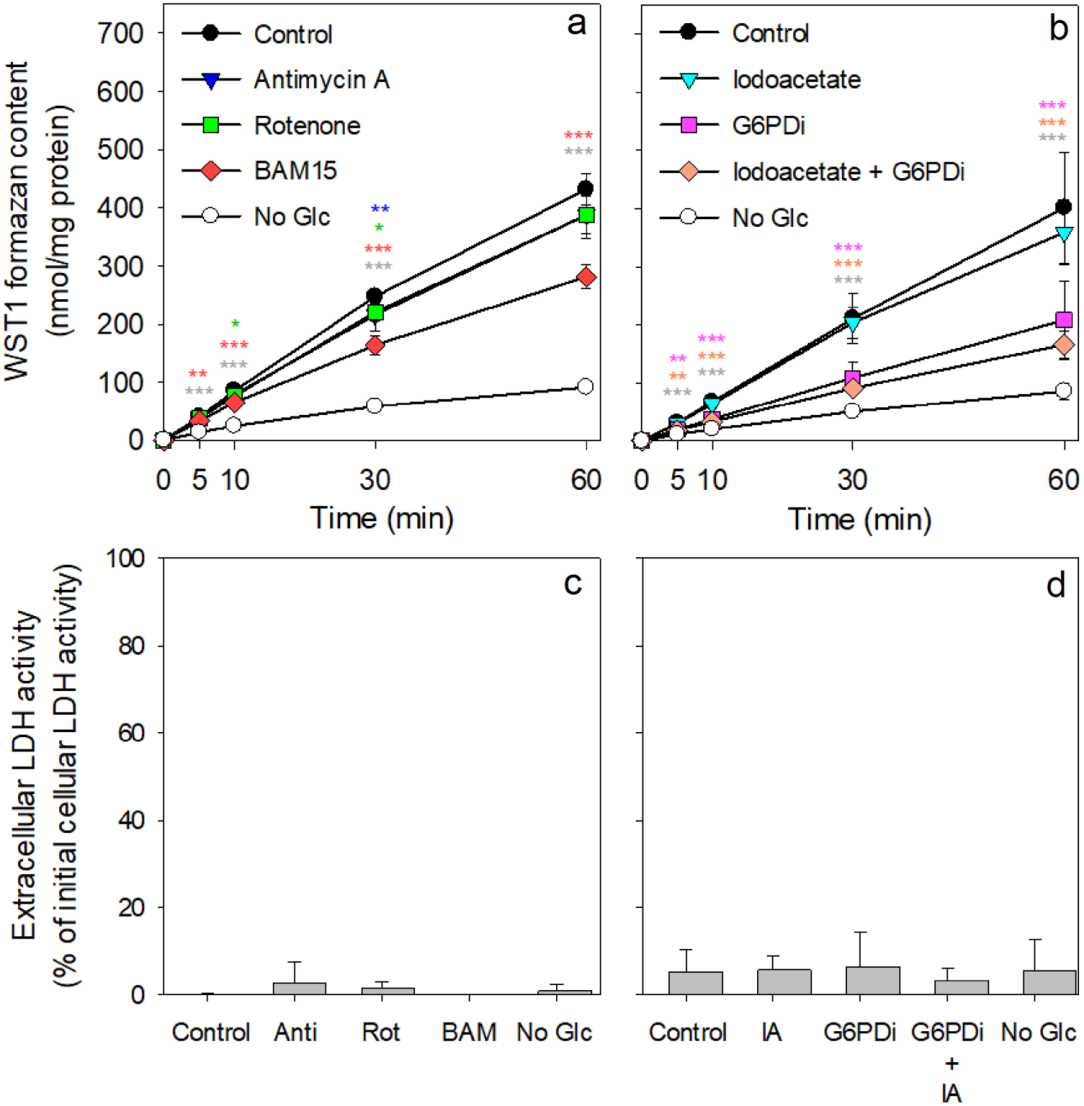
Consequences of the presence of metabolic inhibitors on the β-lapachone-mediated WST1 reduction by astrocytes. The cultures were preincubated for 30 min in the absence of glucose and subsequently incubated in 400 µL IB with 5 mM glucose, 400 µM WST1 and 3 µM β-lapachone in the absence (control) or the presence of 10 µM rotenone (Rot), 10 µM antimycin A (Anti) or 10 µM BAM-15 (BAM; a, c) or in the absence or the presence of 10 µM of the glucose-6-phosphate dehydrogenase inhibitor G6PDi-1 (G6PDi) and/or 0.3 mM of the GAPDH inhibitor iodoacetate (IA; b, d). As further control for a glucose-independent WST1-reduction, an incubation in glucose-free buffer containing WST1 and β-lapachone in the absence of metabolic inhibitors was included (No Glc; open circles). For the given incubation periods, the extracellular WST1 formazan content (**a**, **b**) and for the 60 min incubation the extracellular LDH activity were determined (**c**, **d**). The initial protein contents of the cultures used for the experiments were 106 ± 8 µg per well (**a**, **c**) and 130 ± 18 µg/well (**b**, **d**). The data shown are means ± SD of results obtained in experiments on three independently prepared astrocyte cultures. The significance of differences (as calculated by ANOVA) compared with data for an incubation in the absence of inhibitors (control) is given by *p < 0.05, **p < 0.01, or ***p < 0.001 in the colours indicating the presence of a given inhibitor

### Specific Cellular WST1 Reduction Capacity of Cultured Primary Astrocytes

To determine the basal WST1 reduction capacity of cultured astrocytes we analysed data from a total of 18 experiments (performed on 13 independently prepared cultures) for standard conditions (60 min incubation in 400 µL IB with 3 µM β-lapachone, 400 µM WST1 and 5 mM glucose). The calculated average WST1 reduction per well of the cultures was 47 ± 7 nmol/(well x 60 min), the average protein content of the cultures was 134 ± 24 µg/well and the calculated specific WST1 reduction capacity of the cultures was 362 ± 52 nmol/(mg x 60 min). We did not observe any age-dependent difference in the specific WST1 reduction capacity for the investigated cultures that had an age between 14 and 36 d (data not shown).

### Replacement of Glucose by Other Substrates to Support the β-lapachone-mediated WST1 Reduction in Cultured Astrocytes

To test for the potential of other extracellular substrates to replace glucose as metabolic substrate to provide electrons for the NQO1-mediated WST1 reduction, cultured astrocytes were preincubated for 30 min in glucose-free IB and subsequently incubated for 60 min with β-lapachone and WST1 in the absence or the presence of various potential substrates, including sugars, mitochondrial substrates or amino acids. For all incubation conditions applied, the cell viability was not compromised as demonstrated by the very low activity of extracellular LDH (Table 1). Around 330 nmol/mg of extracellular WST1 formazan was determined for cultures that had been exposed to glucose, while in the absence of metabolic substrate only around 27% (around 90 nmol/mg) of this value were found (Table 1). Of the other substrates investigated, only extracellular mannose was able to fully replace glucose as substrate allowing maximal WST1 formazan generation (Table 1). In contrast, other sugars, mitochondrial substrates or amino acids were not used by cultured astrocytes (values below 27% of glucose control) or only to a low extent (values between 27 and 45% of the glucose control) as metabolic substrates to provide via their metabolism electron for WST1 reduction (Table 1).

**Table 1:** Utilization of extracellular substrates as electron source for β-lapachone-mediated WST1 reduction by astrocytes.

Substrate (5 mM)	LDH (%)	WST1 formazan content	
		(nmol/mg protein)	(%)
No substrate	0 ± 0	88 ± 2	27 ± 1
Glucose	0 ± 0	327 ± 12***	100 ± 4***
Mannose	2 ± 3	338 ± 21***	103 ± 7***
Fructose	1 ± 2	138 ± 7***	42 ± 2***
Galactose	3 ± 1	125 ± 11***	38 ± 3***
Sorbitol	3 ± 5	123 ± 6***	37 ± 2***
Ribose	3 ± 5	111 ± 4*	34 ± 1*
Lactate	2 ± 3	147 ± 11***	45 ± 3***
Pyruvate	2 ± 3	113 ± 6*	34 ± 2*
Alanine	5 ± 7	79 ± 14	24 ± 4
β-Hydroxybutyrate	6 ± 2	102 ± 5	31 ± 2
Acetoacetate	3 ± 3	122 ± 4***	37 ± 1***
Glutamate	2 ± 1	88 ± 13	27 ± 4
Glutamine	6 ± 4	115 ± 4**	35 ± 1**
Glycerol	2 ± 2	102 ± 4	31 ± 1

The cells were pre-incubated for 30 min in glucose-free IB and subsequently incubated in 400 µL IB without (no substrate) or with 5 mM of glucose or of the other substances indicated in the presence of 400 µM WST1 and 3 µM β-lapachone. For the 60 min incubation, extracellular LDH activity (given as percent of the initial cellular LDH activity) and extracellular WST1 formazan content were determined. The initial cellular protein content was 141 ± 8 µg per well. The data shown are means ± SD of results obtained in experiments performed on three independently prepared astrocyte cultures. The significance of differences (as calculated by ANOVA) compared with data for an incubation in the absence of applied substrate in the main incubation is indicated by *p < 0.05, **p < 0.01, or ***p < 0.001

### Effects of Inhibitors of Metabolic Pathways on Astrocytic WST1 Reduction in the Presence of Glucose

Metabolic regeneration of cellular NAD(P)H is essential for continuous β-lapachone-mediated WST1 reduction by astrocytes. To investigate whether cytosolic or mitochondrial processes are involved in providing electrons for β-lapachone reduction, several known inhibitors of metabolic pathways were applied to investigate their potential to lower the cell dependent WST1 reduction (Fig. 4a). None of the applied inhibitors caused any obvious toxicity under the conditions used as demonstrated by the absence of any increase in extracellular LDH activity (Fig. 4c, d). Presence of the mitochondrial respiratory chain inhibitors rotenone [25] or antimycin A [26] did not significantly affect glucose-dependent WST1 reduction by cultured astrocytes, while the presence of the uncoupler BAM-15 [27] lowered WST1 reduction significantly by 35% (Fig. 4a). Also, the GAPDH inhibitor iodoacetate [28] had only a low inhibitory potential and lowered WST1 reduction by 11% (Fig. 4b). In contrast, the pentose-phosphate pathway inhibitor G6PDi-1 [29] had a strong effect on the β-lapachone-mediated WST1 reduction by astrocytes, lowering the total cellular WST1 reduction by around 50% and the glucose-dependent WST1 reduction (difference between values obtained for incubation without and with 5 mM glucose) by around 60% (Fig. 4b). In addition, coincubation of astrocytes with G6PDi-1 plus iodoacetate additively lowered extracellular WST1 formazan generation to around 60% of total WST1 reduction and to around 75% of glucose-dependent WST1-reduction (Fig. 4b).

**Fig. 5:**
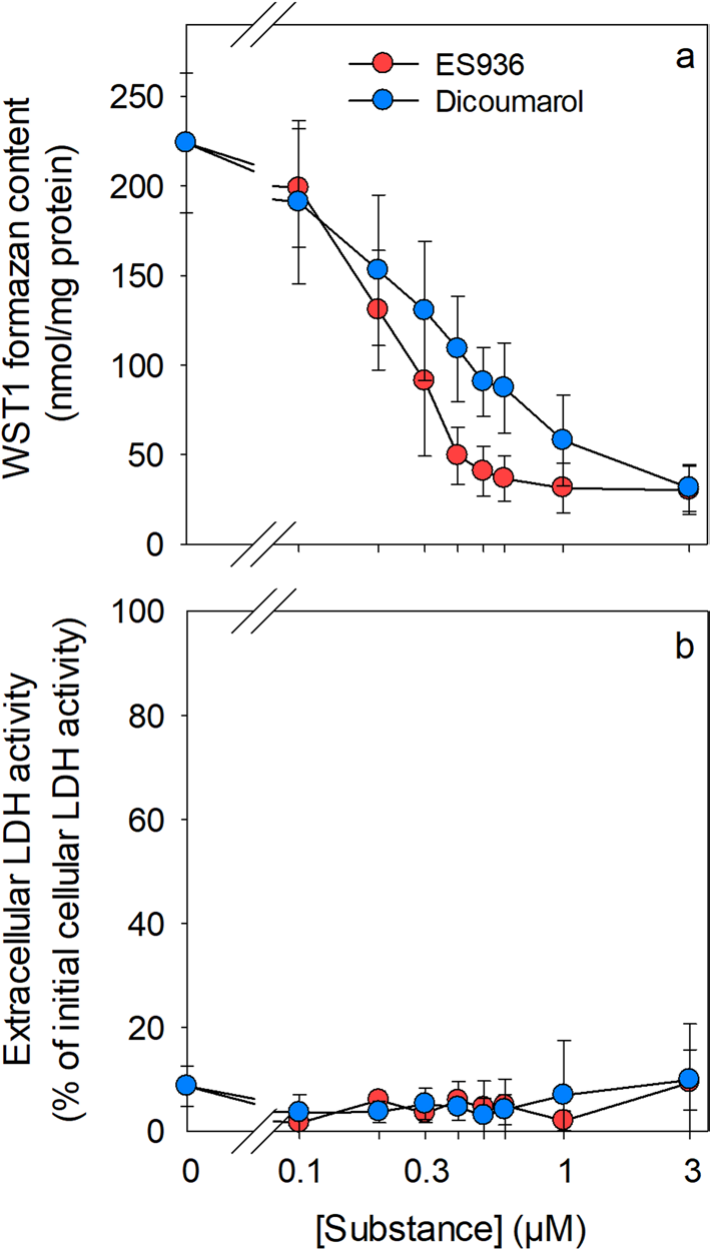
Inhibition of β-lapachone-mediated WST1 reduction by NQO1 inhibitors. Cultured astrocytes were incubated without (0 µM) or with up to 3 µM of the NQO1 inhibitors dicoumarol or ES936 in 200 µL IB in the presence of 5 mM glucose, 400 µM WST1 and 3 µM β-lapachone. The specific extracellular WST1 formazan content (**a**) and the extracellular LDH activity (**b**) were determined for the 60 min incubation. Half maximal inhibitory concentrations were calculated for ES936 (0.22 ± 0.08 µM) and dicoumarol (0.32 ± 0.03 µM). The initial protein content of the astrocyte cultures was 155 ± 15 µg per well. The data shown represent means ± SD of values derived from experiments on three independently prepared astrocyte cultures

### Effects of NQO1 Inhibitors on β-lapachone-mediated WST1 Reduction in Astrocyte Cultures

NQO1 has been reported as the enzyme mainly responsible for the cellular reduction of β-lapachone in cultured astrocytes [7]. In order to test whether the glucose-dependent β-lapachone-mediated WST1 reduction is affected by inhibition of NQO1, cultured astrocytes were incubated in glucose-containing buffer with WST1 and β-lapachone in the absence or the presence of various concentrations of the NQO1 inhibitors ES936 [30] or dicoumarol [31] for 60 min. None of these incubation conditions caused any obvious alteration in cell membrane integrity in the cultures as demonstrated by the absence of any significant increase in extracellular LDH activity (Fig. 5b). Presence of each of the applied NQO1 inhibitors strongly lowered the extracellular accumulation of WST1 formazan in a concentration-dependent manner. The inhibitor concentrations allowing half-maximal extracellular WST1 formazan accumulation within the 60 min incubation period were calculated to be 0.22 ± 0.08 µM for ES936 and 0.32 ± 0.03 µM dicoumarol. In concentrations of 3 µM both inhibitors caused maximal inhibition of the cell-dependent extracellular WST1 reduction (Fig. 5a).

**Fig. 6:**
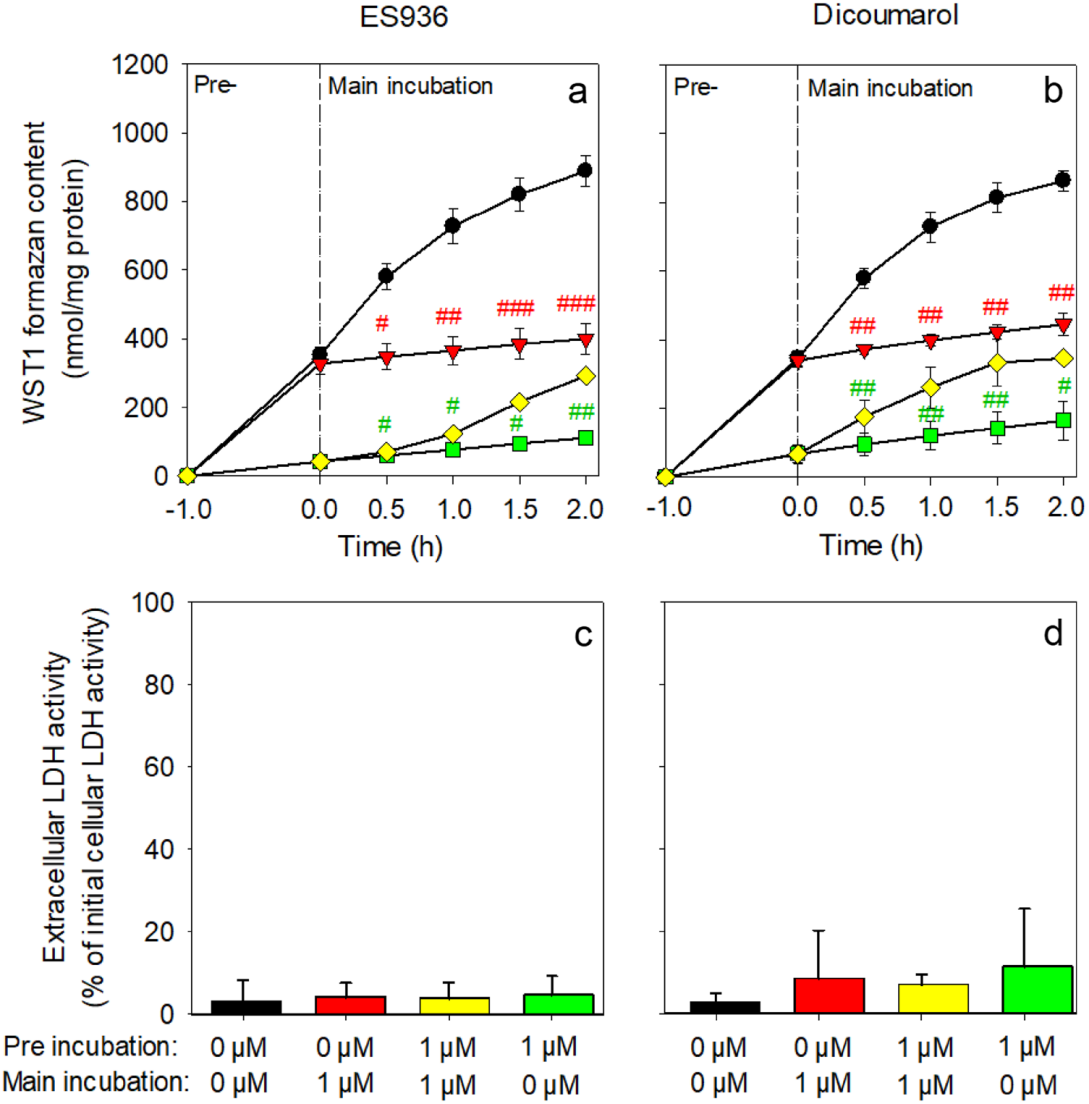
Test for reversibility of NQO1 inhibition by ES936 or dicoumarol. Primary astrocyte cultures were pre-incubated without (0 µM) (a, b; black and red symbols) or with 1 µM ES936 (a; yellow and green symbols) or 1 µM dicoumarol (**b**; yellow and green symbols) in 400 µL IB for 60 min in the presence of 5 mM glucose, 400 µM WST1 and 3 µM β-lapachone. The cells were then washed twice with glucose-containing incubation buffer (5 mM) and incubated without (0 µM) (a, b; black and yellow symbols) or with 1 µM ES936 (a; red and green symbols) or dicoumarol (b; red and green symbols) for up to 2 h in the presence of 400 µM WST1 and 3 µM β-lapachone in 400 µL glucose (5 mM)-containing IB. Extracellular WST1 formazan content (a, b) and extracellular LDH activity (c, d) were determined for the indicated time points (a, b) or after the 120 min main incubation (c, d). The initial protein content of the astrocyte cultures was 173 ± 5 µg per well. The data shown for the main incubation (a, b) are the sum of the values obtained for the 60 min preincubation plus the values for the indicated time points of the main incubation. The data represent means ± SD of values from experiments performed on three independently prepared astrocyte cultures. The significance of differences (calculated by the paired t-test) between data for incubations without (0 µM) and with 1 µM ES936 or 1 µM dicoumarol in the main incubation are indicated by ^#^p < 0.05, ^##^p < 0.01 or ^###^p < 0.001

To test for the reversibility of the NQO1 inhibitor-mediated impairment of cell-dependent WST1 reduction, astrocytes were preincubated without or with either 1 µM ES936 or dicoumarol for 60 min, washed and subsequently incubated for up to 120 min in the presence or the absence of the inhibitors (Fig. 6). None of these incubations caused any obvious release of cellular LDH within the main incubation period (Fig. 6c, d). Presence of each of the inhibitors drastically lowered WST1 formazan accumulation during the preincubation as well as during the main incubation of cells that had been preincubated without or with an inhibitor (Fig. 6a, b). In contrast, for cells that had been preincubated with an NQO1 inhibitor, extracellular WST1 formazan accumulation was re-established during the main incubation after removal of the inhibitor (Fig. 6a, b), demonstrating reversibility of the NQO1-inhibition by ES936 and dicoumarol in astrocyte cultures.

## Discussion

The NQO1 substrate β-lapachone in a concentration of 20 µM has been recently reported to act as membrane permeable electron cycler in cultured astrocytes that mediates transfer of electrons from intracellular metabolic sources for extracellular reduction of the membrane-impermeable tetrazolium dye WST1 [7]. However, the high concentration of 20 µM β-lapachone also causes oxidative stress and impairs cytosolic lactate production [7]. These adverse consequences of a treatment of astrocytes with 20 µM β-lapachone were abolished by lowering the concentration of β-lapachone to 3 µM, while efficient extracellular WST1 reduction in the presence of WST1 was still detectable for this low β-lapachone concentration. Therefore, it was concluded that presence of 3 µM of the redox cycler β-lapachone is suitable to monitor via the NQO1-dependent extracellular WST1 accumulation the intracellular pathways of astrocytes that contribute to the regeneration of NADH and NADPH. Nevertheless, as the extracellular WST1 reduction by cell-derived β-lapachol is at least partially prevented by superoxide dismutase [7], it has to be expected that also in the presence of 3 µM β-lapachone some superoxide is generated by the spontaneous autoxidation of the β-lapachol generated in the NQO1-catalysed reaction. However, in the cells this appears not to cause detectable oxidative stress as indicated by the absence of any GSSG accumulation. The known high antioxidative potential of astrocytes [9, 32] appears to deal very well with the low amounts of superoxide that are likely to have been generated by intracellular autoxidation of β-lapachol during an incubation with 3 µM β-lapachone. As astrocytes express superoxide dismutases [33] the superoxide derived from β-lapachol will be disproportionated to oxygen and H_2_O_2_ and the latter will be efficiently removed by the high capacity of astrocytes to clear the peroxide via both catalase and glutathione-dependent pathways [34]. Only for concentrations of β-lapachone above 3 µM, this antioxidative potential of astrocytes appears to be insufficient to prevent oxidative stress, as demonstrated by cellular GSSG accumulation, intracellular ROS detection, impaired glycolysis and cell toxicity [7]. Interestingly, the potential of β-lapachone to compromise cell viability of cultured astrocytes appears to be much lower in the presence of WST1 (present report) compared to incubations without WST1 [7], which may be due to the antioxidative potential of WST1 as scavenger of extracellular superoxide under the conditions used.

The extracellular WST1 reduction in the presence of 20 µM [7] or 3 µM (present report) β-lapachone requires active NQO1 in astrocytes, as demonstrated by the strong inhibition of astrocytic WST1 formazan generation in the presence of the known NQO1 inhibitors dicoumarol [31, 35] and ES936 [30]. Both inhibitors lowered cell-dependent WST1 reduction by 50% at inhibitor concentrations of around 0.3 µM as expected for inhibitors that have a K_i_ for NQO1 in the nanomolar range [4].

The inhibition by dicoumarol or ES936 of NQO1-dependent astrocytic WST1 reduction was reversed by washing the cells, demonstrating reversibility of the inhibitory action of both inhibitors for the test system used. This was expected for a dicoumarol treatment as dicoumarol in considered as a competitive NQO1 inhibitor [6, 36, 37], but unexpected for an ES936 treatment as ES936 has been reported as irreversible NQO1 inhibitor [30]. Thus, the NQO1 in rat astrocytes may be less responsive to the proposed [5] covalent modification of NQO1 by ES936, at least under the experimental conditions used for the present report.

The almost linear increase in astrocytic WST1 reduction after application of 3 µM β-lapachone was used to investigate which cellular metabolic pathways may be involved in providing the reduction equivalents for the NQO1 in astrocytes. Even in the absence of glucose an increase in extracellular WST1 formazan was observed during a 60 min incubation that accounted for around 44% of the values determined for glucose-fed cells. This WST1 reduction in glucose-free incubation buffer was lowered by a 30 min preincubation of the cells in glucose-free medium, suggesting that the cells generated substantial amounts of the NAD(P)H used for NQO1-dependent WST1 reduction by the metabolism of substrates and metabolic intermediates that had been present in the cells at the onset of the glucose-free incubation, as also reported for incubations of astrocytes with menadione as redox cycler [6]. These substrates are likely to include free glucose in the cells that is derived from the initial high glucose concentration present in the culture medium [38] and cellular glycogen that is rapidly mobilised after glucose deprivation in astrocytes [39].

The NQO1-dependent WST1 reduction in astrocytes strongly depended on the concentration of glucose applied with half-maximal and maximal effects observed at initial glucose concentrations of 0.1 and 0.5 mM, respectively. Glucose is taken up into astrocytes mainly via the glucose transporter GLUT1 [40], is subsequently phosphorylated by hexokinase which has a very low K_M_ value of around 30 µM for glucose [41] and is further metabolised mainly by glycolysis and PPP, pathways which provide electrons as NADH or NADPH, respectively [10, 20, 42]. As astrocytes are considered as glycolytic cell type that efficiently metabolises glucose [43], efficient WST1-reduction was expected for glucose-treated astrocytes. Among the other substrates applied to test as potential glucose substitutes to supply electrons for NQO1-dependent WST1 reduction, only mannose but not the other sugars were found to be efficiently metabolised to provide electrons for WST1 reduction, consistent with literature data on the ability of cultured astrocytes to metabolise such extracellular substrates [6, 36, 39, 44–46]. Also astrocytic substrates that are mainly metabolized in mitochondria such as lactate, pyruvate, ketone bodies and amino acids [47, 48] were at best to a low extent able to provide electrons for NQO1-dependent WST1 reduction. Among those mitochondrial substrates, lactate appeared to be the best, enabling around 25% of the glucose-dependent WST1 reduction. Most likely the NADH generated by lactate oxidation via cytosolic LDH contributes to the observed low NQO1-dependent WST1 reduction in lactate-exposed astrocytes. In contrast, mitochondrial NAD(P)H generating processes appear to provide only little NAD(P)H for the cytosolic NQO1 to enables β-lapachone-mediated WST1 reduction. This view is supported by the rather low potential of inhibitors of mitochondrial respiration to inhibit glucose-dependent WST1 formation. Of the mitochondrial modulators investigated only the uncoupler BAM-15 [27] lowered the glucose-dependent WST1 reduction substantially by around 44%. This lowered WST1 reduction could be the consequence of a reduced availability of cytosolic NADH for NQO1 due to an accelerated shuttling of cytosolic electrons [49] into uncoupled mitochondria.

The data obtained by experiments using extracellular substrates as potential glucose substitutes and mitochondrial inhibitors support the view [6] that predominately cytosolic pathways are providing the NAD(P)H for cytosolic NQO1-dependent reduction processes [6]. NQO1 accepts electrons from both NADH and NADPH for the reduction of its substrates [16, 50] and similar K_M_ values of astrocytic NQO1 for the substrates NADH and NADPH were reported [6]. However, in the presence of the glucose-6-phosphate dehydrogenase inhibitor G6PDi-1 [29] around 60% of the glucose-dependent β-lapachone-mediated WST1 reduction was prevented, while the GAPDH inhibitor iodoacetate [28] had only little additive inhibitory potential on astrocytic WST1 reduction. This strongly suggests, that for astrocytes PPP-generated NADPH, and not glycolysis-derived NADH, is the preferred electron source for cytosolic NQO1-catalysed reductions.

For our study we have used inhibitors of enzymes to identify metabolic pathways that may be involved in providing electrons via NADH or NADPH for the NQO1-dependent reduction of β-lapachone in astrocytes that is finally monitored by quantifying extracellular WST1 formazan accumulation. For such data it is important to consider the specificity of the inhibitors used in order to avoid misinterpretation of the results obtained. The natural compounds rotenone and antimycin A are frequently used as inhibitors for complex I and complex III of the respiratory chain, respectively, but extended exposure to such inhibitors can also cause some direct or indirect side effects [51, 52]. However, such side effect would not explain the absence of an inhibitory potential of these inhibitors on astrocytic WST1 reduction. Both inhibitors have been found to impair in the concentrations applied the mitochondrial membrane potential of astrocytes [48] (data not shown) and stimulate glycolytic lactate production in astrocytes [46, 53], demonstrating their potential to impair mitochondrial respiration for the conditions used. Thus, the inability of rotenone and antimycin A to severely lower astrocytic WST1 reduction demonstrates that mitochondrial processes do not provide substantial amounts of electrons for astrocytic NQO1. This is consistent with the almost exclusive cytosolic localization of NQO1 in astrocytes [6].

The data obtained for iodoacetate-treated astrocytes should be carefully interpreted as this compound is known to react with activated thiol groups which may cause unwanted side effects. However, iodoacetate in the concentration applied in our study has previously been reported to efficiently inactivate GAPDH activity in cultured astrocytes, while having no effect on several other enzymes, including G6PDH [28]. In the current study iodoacetate only slightly lowered glucose-dependent WST1 reduction and this inhibition was additive to the strong inhibition found for the G6PDH inhibitor. If iodoacetate would have acted under the conditions used via a potential side effect on one of the main players that are involved in astrocytic WST1 reduction, such as the NQO1 activity or the cellular NADPH regeneration via the PPP, a strong impairment of WST1 reduction should have been the consequence, which was not observed.

G6PDi-1 has only very recently been reported as efficient inhibitor of G6PDH [29] and unspecific actions of this compound on other metabolic pathways have to our knowledge not been reported so far. Preliminary experiments revealed that at least the glycolytic glucose consumption and lactate production by cultured astrocytes is not affected for many hours in the presence of G6PDi-1 (data not shown), supporting our interpretation that mainly PPP-derived NADPH provides the electrons for cytosolic NQO1 in astrocytes.

In conclusion, β-lapachone in a low concentration of up to 3 µM can be used as suitable redox cycler to monitor by quantification of extracellular WST1 formazan accumulation the cytosolic pathways that provide NADH and NADPH for cytosolic NQO1. The established optimised conditions avoid the oxidative stress that occurs in cultured astrocytes after exposure to higher concentrations of β-lapachone [7] or menadione [19], thereby minimizing a potential interference of the applied indicator system with oxidative stress-induced alterations in metabolism. In addition, the experimental settings applied could be useful for further studies on the cytosolic regeneration of NADPH which appears to be the main intracellular source of electrons for NQO1-catalysed reduction processes in cultured astrocytes. A limitation of our study is that the contribution of different metabolic pathways was exclusively studied by pharmacological inhibition of crucial enzymes of these pathways. Although high selectivity of most of the frequently applied inhibitors can be assumed, potential side effects cannot be fully excluded. Experiments using knockdown or knockout approaches for given enzymes in cultured astrocytes could be useful to confirm the finding that mainly PPP-derived NADPH is used in astrocytes to supply NQO1 with electrons. It would also interesting to test for potential long-time consequences of an exposure of astrocytes to 3 µM β-lapachone in the presence or the absence of WST1. Targets of such studies could be a potential delayed cell toxicity as well as a potential adaptation of the cells to the mild ROS load generated by NQO1 plus 3 µM β-lapachone, for example an upregulation of NADPH-regenerating and/or antioxidative enzymes.

## Data Availability

Enquiries about data availability should be directed to the authors.
